# Combination of Metformin and Sorafenib Induces Ferroptosis of Hepatocellular Carcinoma Through p62-Keap1-Nrf2 Pathway

**DOI:** 10.7150/jca.76618

**Published:** 2022-09-06

**Authors:** Kezhong Tang, Qing Chen, Yanmo Liu, Lantian Wang, Wenjie Lu

**Affiliations:** 1 Department of Surgery, The Second Affiliated Hospital, Zhejiang University School of Medicine, #88 Jiefang Road, Hangzhou 310009, PR China.; 2 Department of Pharmacy, Affiliated Sir RunRun Shaw Hospital, Zhejiang University School of Medicine, Hangzhou 310009, PR China.

**Keywords:** Hepatocellular carcinoma (HCC), sorafenib, metformin, ferroptosis, NF-E2-related factor 2 (Nrf2)

## Abstract

Hepatocellular carcinoma (HCC) is one of the most lethal cancers in the world. Sorafenib is the first small-molecule multi-kinase inhibitors approved by FDA for treatment of advanced HCC. Metformin has been demonstrated to have benefit for preventing cancer progression. In human recurrent HCCs, NF-E2-related factor 2 (Nrf2) was overexpressed and associated with poor survival. Nrf2 related signaling pathway plays central role to mediate cellular resistance to sorafenib through protecting HCC cells from ferroptosis. The effect of Combination treatment for HCC cells and the intrinsic mechanism have not been reported. In this study, metformin augmented the anti-tumor effect of sorafenib for HCC through ferroptosis induction by inhibiting Nrf2 related pathway. Based on the results of Nrf2 knockdown and p62 knockdown study, the combination of sorafenib and metformin suppressed proliferation of HCC cells through p62-Keap1-Nrf2/HO1 signaling way. Size of xenografts treated with the combination of sorafenib and metformin was smaller than other groups *in vivo*. Moreover, the combination treatment greatly induced ferroptosis in HCC cells through inhibiting Nrf2 expression. Based on our findings, the combination treatment suppressed proliferation of HCC cells through ferroptosis induction, by p62-Keap1-Nrf2/HO1 signaling way.

## Introduction

Hepatocellular carcinoma (HCC) is the fifth most commonly diagnosed cancer and the second cause of cancer-related mortality in the world 1. Late diagnosis and quickly drug resistance to chemotherapy are the main reasons for poor result of HCC patients. Sorafenib is the first small-molecule multi-kinase inhibitors approved by FDA for treatment of advanced HCC 2. However, sorafenib is proved to only extend the life expectancy of patients with HCC by a few months because of quickly acquired drug resistance attributed to the acquired or intrinsic resistance of cancer cells to apoptosis 3, 4.

Recently, sorafenib was recognized as an inducer of ferroptosis, a new type of non-apoptotic cell death found and named by Stockwell et al. 5 in 2012, defined as an iron-catalyzed form of regulated necrosis that occurs through excessive peroxidation of polyunsaturated fatty acids (PUFAs) 6-8. Compared with other types of cell death influenced by caspases activity directly or indirectly, ferroptosis seems to have little known molecular cross-talk to other types of cancer death 9. The emerging evidences up to now indicate patients could still get benefits from ferroptosis induction treatment who failed in apoptosis and necroptosis induction treatment 10. Therefore, targeting sorafenib-induced ferroptosis may represent an effective therapy for better treatment of HCC.

Intrinsic and adaptive antioxidant accumulation is the main mechanism helps cancer cells escape from oxidative damage 11. Nuclear factor E2-related factor 2 (Nrf2) plays central role to regulate antioxidant enzyme genes, reduce ROS productions and defend against oxidative stress. At the same time, Nrf2 was also demonstrated to regulate cell ferroptosis. Under homeostatic conditions 12, 13, Nrf2 locates in the cytoplasm at low levels and inhibited by kelch-like ECH-associated protein 1 (KEAP1)-mediated ubiquitination and proteasomal degradation. Intrinsic and adaptive oxidative stress stabilizes cytoplasmic Nrf2 and translocates Nrf2 to the nucleus to promote cytoprotective target genes transcription. p62 is the upstream of P62-Keap1-Nrf2 pathway and can recruit Keap1 from the Nrf2-Keap1 complex, sequester Keap1 into autophagosomes, transfer Nrf2 into the nucleus and regulate antioxidative stress genes 14. Thus, p62-Keap1-Nrf2 pathway plays a critical role in ferroptosis induction. Nrf2 is frequently found to be up regulated in HCC tissues and its expression is associated with drug resistance and poor prognosis 15. Therefore, targeting Nrf2 may be an effective way to induce sorafenib-induced ferroptosis and enhance tumor therapy.

Metformin, which is widely used as anti-diabetic drug, has demonstrated important anti-cancer effects both *in vitro* and *in vivo* in many studies 16-18. Metformin combination with other treatment strategies has also been widely reported 19. Metformin was demonstrated to enhance anti-cancer treatments for different cancer cell types through various mechanisms including mitochondrial apoptosis induction and inhibition of DNA synthesis 20. Several studies have demonstrated the close connection between metformin and Nrf2 to overcome chemotherapy resistance. Although the signal transduction mechanisms of detoxification of reactive oxygen species (ROS) have been explained in some studies, few studies have focused on the connection between metformin and ferroptosis, which may be a new direction to sensitize chemotherapy 21, 22.

In this study, we found that overexpression of Nrf2 protein in HCC tissues was associated with poor patient survival. A further investigation on the potential mechanism of metformin inhibiting the antioxidative capacity and inducing ferroptosis in HCC cells in the context of sorafenib treatment was performed.

## Materials and Methods

### Drugs and reagents

Assay kits for the detection of cell survival (CCK8 kit) was purchased from Beyotime (Shanghai, China). GSH and MDA were obtained from MedChemExpress (Shanghai, China). Sorafenib, Ferrostatin-1 and Liproxstatin-1 were purchased from Selleck Chemicals (Shanghai, China). ZVAD-FMK, Necrostatin and Necrosulfonamide were obtained from Sigma-Aldrich (Shanghai, China). Metformin was obtained from aladdin (Shanghai, China). Antibodies against, including Nrf2, HO-1, P62, Keap1 and β-actin, were obtained from MedChemExpress (Shanghai, China). Nrf2 siRNA (GTTGCCCACATTCCCAAATCA) and P62 siRNA (GAACAGAGCGGGATCAAGAAT) were designed and constructed by QIAGEN (Shanghai, China).

### Patient and Specimens

The inclusion and exclusion criteria for patients included in the study was as follows: Inclusion criteria: 1. Patients with age ≤ 80. 2. Patients diagnosed as hepatocellular carcinoma by images or biopsy. 3. Patients received radical resection before HCC recurrence. Exclusion criteria: 1. Patients refused sorafenib therapy. 2. Patients with serious primary disease except HCC.

Tissue specimens including HCC and adjacent noncancerous liver tissue were obtained at the time of operation at the Second affiliated hospital of Zhejiang University of Medicine from 2016-2019. The use of human clinical specimens was approved by the Institutional review board of the Second Affiliated Hospital of Zhejiang University of Medicine (No. 2021-0256). This work was a retrospective study without the requirement of written informed consent.

### Immunohistochemistry staining and scoring standard

Immunohistochemical analysis of paraffin-embedded tissue sections was performed as previously described 23, 24. The serial sections were stained with hematoxylin eosin (HE) and immunohistology for the determination of the Nrf2 expression level (1:200; Santa Cruz, CA) in clinical HCC samples. The protein expression level was assessed by Mean of Integrated Option Density (IOD) with Image-ProR Plus. All of the Immunohistochemical sections were photographed for three yields in the same standard. After that, we selected Area of Interesting (AOI) and detected IOD to gain Mean of IOD (IOD/AOI, MI). The Mean of MI must be normalized to positive control (vascular smooth muscle cells). Tissues were divided into high and low group according to Mean of MI of Nrf2 expression level.

### Cell Culture

The HepG2 and HUH-7 human HCC cell lines were used in this study. HCC cell lines were cultured at 37 °C in a humidified atmosphere with 5% CO2. RPMI-1640 supplemented with 10% fetal bovine serum (FBS), 1% penicillin streptomycin and 1 mM sodium pyruvate was used to culture these cells.

### Cell Viability Assay

Cell viability was performed with CCK8 kit according to the instruction supplied by manufacture. Cells were seeded in 96-well plates with a density of 3*10^3^ cells/well overnight and cotreated with various concentrations of sorafenib and metformin. Then, 10ul CCK8 reagent was added to each well. Cells were incubated at 37 °C for 1 h for further evaluation. The absorbance at 450 nm was measured by microplate reader according to the instruction. The cell viability in each group was calculated and the ratio to vehicle control was obtained.

### Iron Assay

Iron Assay Kit was used to evaluate the relative iron concentration in cell lysates according to instructions supplied by manufacturer.

### Lipid Peroxidation Assay

The Lipid Peroxidation (MDA) Assay Kit was used to evaluate the relative malondialdehyde (MDA) concentration in cell lysates according to instructions supplied by manufacturer.

### Glutathione Assay

The Glutathione Assay Kit was used to evaluate the relative glutathione (GSH) concentration in cell lysates according to instructions supplied by manufacturer.

### Cytoplasmic and nucleus protein extraction

The Nuclear and Cytoplasmic Protein Extraction kit (Thermal Scientific, USA) was used to prepare nucleic and cytosolic fractions. Briefly, the collected cells were first cultured in cold hypotonic buffer on ice for 50 min. Then, supernatant was obtained after centrifugation at 12000 g for 5 min (cytosolic fraction). For nuclear fraction, the extracts were washed and resuspended in lysis buffer. Supernatant was obtained after centrifugation at 12000 g for 5 min.

### Western blot

Cells were first lysed in RIPA buffer supplemented with protease and phosphorylation inhibitor cocktail. BCA Protein Assay Kit was used to evaluate protein concentration according to instructions supplied by manufacturer. Samples were then separated by SDS-PAGE. After that, proteins were transferred onto PVDF membranes, blocked with 5% milk-TBST, and incubated with the primary antibodies overnight. PVDF membranes were then washed with TBST for three times. After that, the membranes were incubated with horseradish peroxidase 25-conjugated secondary antibodies. ECL Plus chemiluminescence detection kit was used to visualize HRP after washing three times with TBST. The signals were detected by Gel Imager (Bio-Rad).

### Real-time quantitative polymerase chain reaction (RT-qPCR)

TRIzol was used to isolate RNA from cells according to instruction supplied by manufacturer. Nanodrop 1000 (Thermo Fisher) was used to quanify RNA. Prime-Script^TM^ RT reagent Kit (TAKARA, China) was used to perform reverse transcription according to the protocol supplied by the manufacture. TransStart® Green qPCR SuperMix (TransGen Biotech, China) was used for quantitative PCR according to the protocol supplied by the manufacture. β-actin were worked as the housekeeping gene. The primers for qPCR are shown below: NRF2, forward, TCCAGTCAGAAACCAGTGGAT; reverse: GAATGTCTGCGCCAAAAGCTG; HO-1, forward: AAGACTGCGTTCCTGCTCAAC; reverse: AAAGCCCTACAGCAACTGTCG. 2^-ΔΔCt^ method was used to calculate the relative expression of RNA. The amplification efficiency varied between 90% and 105%.

### Animal experiments

BALB/C nude mice (aged 5 weeks) were obtained from Laboratory Animal Center in Zhejiang University. These mice were first incubated in SPF condition for one week. After that, xenografts were finished by subcutaneously injecting HepG2 cells (2*10^6^) into the right flanks of BALB/C nude mice. These mice were randomly divided into 4 groups when the size of tumors reached 100-200 mm^3^. For sorafenib treated group, mice were given oral administration of sorafenib according to 10 mg/kg every day. For metformin treated group, mice were given oral administration of metformin according to 150 mg/kg every day. For the combined therapeutic experiments, sorafenib and metformin were given to mice at the same time. The change of tumor volumes and body weight were recorded every 3 days in the study.

### Statistical analysis

SPSS (version 26.0) was used to perform all statistical calculations. All Data was presented as mean ± standard deviation 9 according to the results of three independent experiments. The student's t-test was used to analyze the differences between two groups. One-way analysis of variance (ANOVA) was used to compare the differences among multiple groups. The standard of significance was defined as *p*<0.05. GraphPad Prism 8.0 software (GraphPad Software, La Jolla, CA, USA) was used to construct all histograms and curves.

## Results

### Nrf2 was overexpressed in human HCC specimens

**Figure 1:**
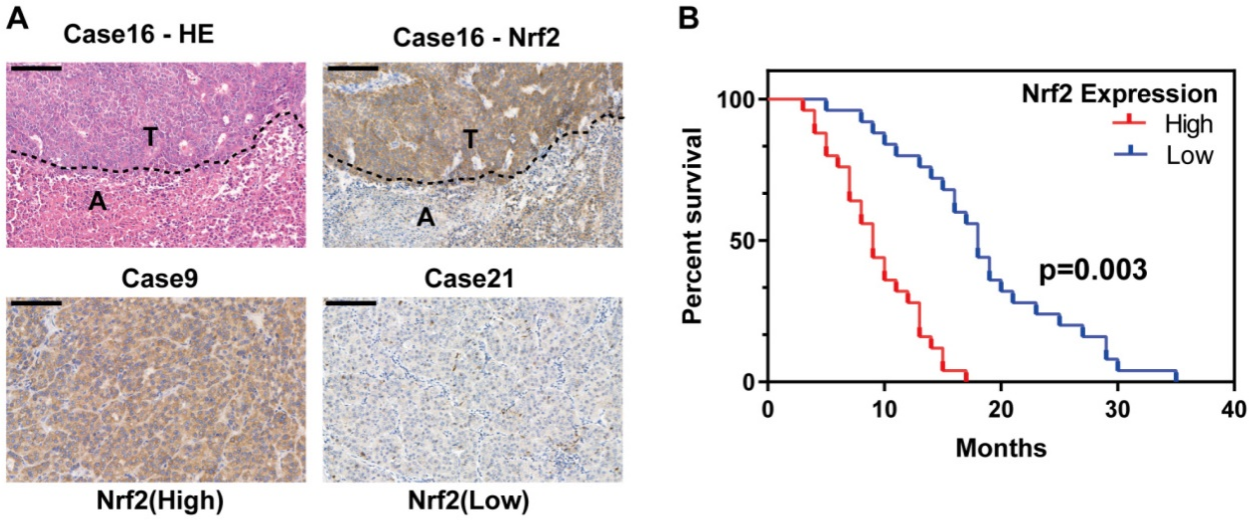
** Nrf2 is activated in HCC population and contributes to poor patient survival. A** Tissue microarray consisting of 50 tumor tissues and corresponding nontumor liver tissues was subjected to HE and IHC analysis. Representative HE and IHC images show Nrf2 expression in HCC and its corresponding nontumor counterpart (case 16). Two HCC cases, one with high Nrf2 expression (case 9) and the other with low Nrf2 expression (case 21), are shown. Scale bar represent 20 um. **B** The overall survival rate of HCC patients after tumor recurrence. Patients with high Nrf2 overexpression were with significantly lower OS compared with those with low Nrf2 expression (*p*=0.003).

Nrf2 protein expression is reported to be associated with poor survival of HCC patients. To further evaluate the expression of Nrf2 in HCC tissues, a tissue microarray consisting of 50 HCC samples containing nontumor liver tissues were performed and evaluated by hematoxylin-eosin and immunohistochemical staining. Tissues were divided into high Nrf2 expression group and low Nrf2 expression group depending on the upregulation of Nrf2 in tumor tissue compared with nontumor tissue (Figure 1A). The basic characteristics of patients involved were shown in Table 1. The results showed high Nrf2 expression was associated with shorter overall survival (p=0.03) (Figure 1B).

### Metformin augmented the cytotoxicity of sorafenib in HCC cells

**Figure 2:**
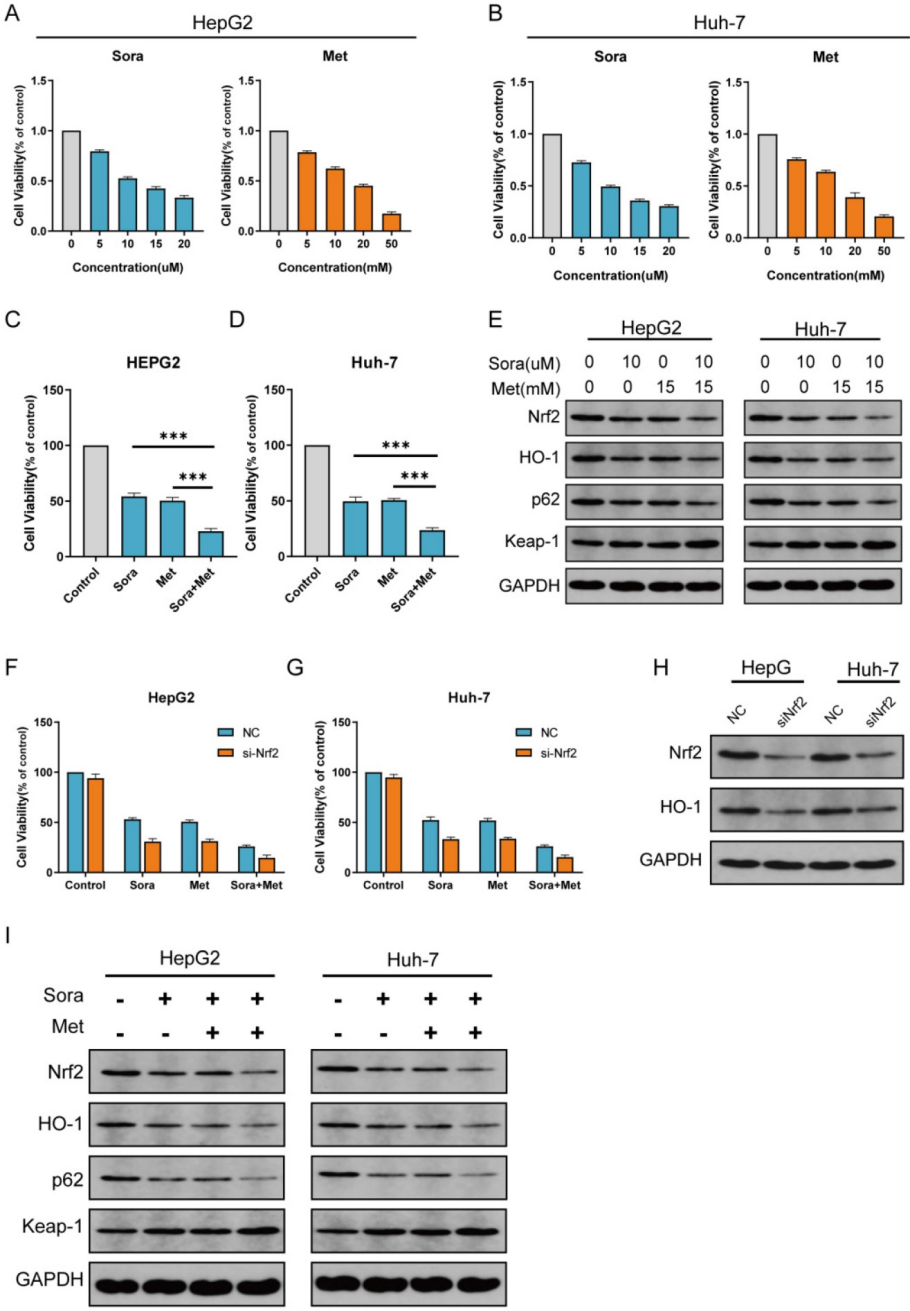
** Combination treatment with metformin and sorafenib synergistically suppresses proliferation in HCC cells through inhibiting NRF2 expression. A** HepG2 cells were treated with different concentrations of sorafenib (IC50=11.04 uM) and metformin (IC50=16.69 mM). **B** Huh-7 cells were treated with different concentrations of sorafenib (IC50=9.63 uM) and metformin (IC50=15.34 mM). **C** HepG2 cells were co-treated with sorafenib (10 uM) and metformin (15 mM). **D** Huh-7 cells were co-treated with sorafenib (10 uM) and metformin (15 mM). **E** combination treatment synergistically inhibited P62-Keap1-Nrf2/HO-1 signal way in HepG2 and Huh-7 cells. **F** Nrf2 knockdown HepG2 cell was significantly inhibited by sorafenib and metformin compared with control group. **G** Nrf2 knockdown Huh-7 cell was significantly inhibited by sorafenib and metformin compared with control group. **H** Protein levels of Nrf2 and HO-1 were assayed with western blot for control group and Nrf2 knockdown group. I Protein levels of P62, Keap1, Nrf2 and HO-1 were assayed with western blot for Nrf2 knockdown group treated with sorafenib (10 uM) and metformin (15 mM).

The comparison of *in vitro* cytotoxicity of sorafenib and metformin in two human HCC cell lines was performed. Cells co-treated with sorafenib (0-20uM) and metformin (0-50mM) for 24 h. Different dose-dependent degrees of cytotoxicity was shown in Figure 2A, B. The level of IC50 was calculated from the cell viability assay. Then, cells were co-treated with sorafenib (10 uM) and metformin (15 mM), which significantly suppressed proliferation in HCC cells (Figure 2C, D).

### Inhibiting Nrf2 expression was the main mechanism for the combination treatment to suppress HCC cells proliferation

Since Nrf2 oriented signal way is strongly associated with resistance to sorafenib, and metformin is closely related to inhibit Nrf2 expression, we then evaluated Nrf2 expression in the two human HCC cell lines (Figure 2E). Nrf2 expression was significantly inhibited when co-treated with sorafenib and metformin. Nrf2 expression level is closely related to the proliferation of HCC, which indicated the close relationship to the co-treatment in the study.

To determine the role of Nrf2 during the treatment in this study, Nrf2 knockdown cells were cultured (Figure 2H). Compared with HepG2 and Huh-7 cells, Nrf2 knockdown HepG2 and Huh-7 cells were more sensitive to sorafenib, metformin and combined treatment (Figure 2F, G). Protein level of Nrf2 was consistent with proliferation of Nrf2 knockdown HCC cells in the study (Figure 2I).

### P62-Keap1-Nrf2/HO-1 signal way was the main mechanism for the combination treatment to suppress HCC cells proliferation

**Figure 3:**
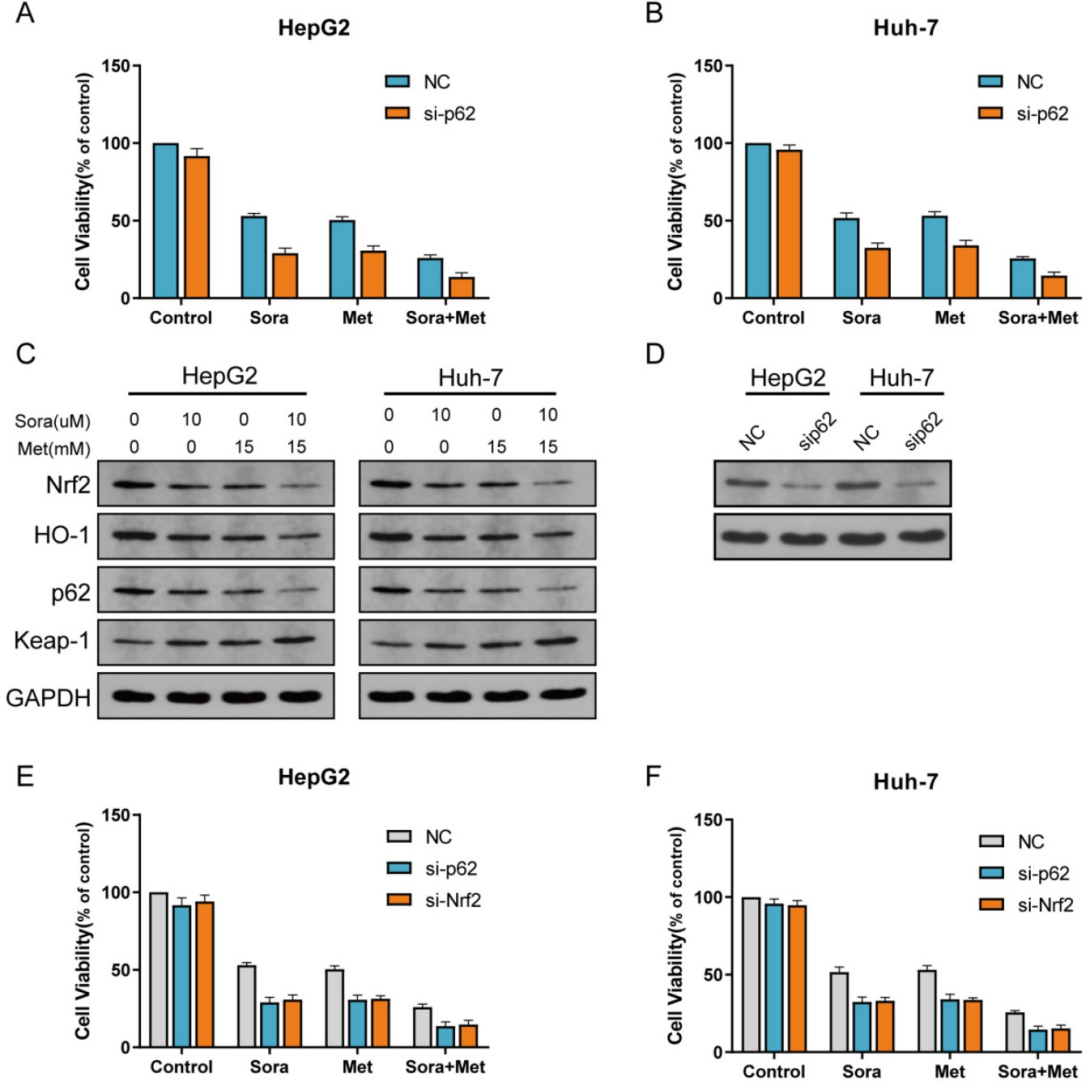
** The combination treatment suppresses proliferation in HCC cells through P62-Keap1-Nrf2 signal way. A** P62 knockdown HepG2 cell was significantly inhibited by sorafenib and metformin compared with control group. **B** P62 knockdown Huh-7 cell was significantly inhibited by sorafenib and metformin compared with control group. **C** Protein levels of Nrf2, HO-1, P62 and Keap1 were assayed with western blot for P62 knockdown group treated with sorafenib (10 uM) and metformin (15 mM). **D** Protein level of P62 was assayed with western blot for control group and P62 knockdown group.** E** P62 knockdown HepG2 cell has similar reaction to sorafenib and metformin compared with Nrf2 knockdown group. Both were significantly inhibited by sorafenib and metformin compared with control group. **F** P62 knockdown Huh-7 cell has similar reaction to sorafenib and metformin compared with Nrf2 knockdown group. Both were significantly inhibited by sorafenib and metformin compared with control group.

P62-Keap1-Nrf2 signal way is the main mechanism to modulate Nrf2 expression in HCC cells. HO-1 is the main protein to modulate ROS reaction which is the downstream of Nrf2. To determine the role of P62-Keap1-Nrf2/HO-1 signal way in combination treatment in the study, protein expression of P62, Keap1 and HO-1 was obtained with western blot for normal group and Nrf2 knockdown group treated with sorafenib, metformin and combined (Figure 2E, I). The expression levels of P62, Keap1 and HO-1 were consistent with that of Nrf2, which indicated P62-Keap1-Nrf2/HO-1 signal way was the possible mechanism for the combination treatment to suppress HCC cells proliferation. Then more, P62 knockdown HCC cells were cultured (Figure 3D). Compared with HepG2 and Huh-7 cells, P62 knockdown HepG2 and Huh-7 cells were more sensitive to sorafenib, metformin and combined treatment (Figure 3A, B). Protein levels of Nrf2, HO-1, P62 and Keap1 were assayed with western blot for P62 knockdown group treated with sorafenib and metformin, which was consistent with Nrf2 knockdown group (Figure 3C). P62 knockdown groups has similar reaction to sorafenib and metformin compared with Nrf2 knockdown group. Both were significantly inhibited by sorafenib and metformin compared with control group (Figure 3E, F). This result indicated P62-Keap1-Nrf2/HO-1 signal way was the main mechanism for the combination treatment to suppress HCC cells proliferation.

### The combination treatment suppressed HCC cells proliferation through inhibiting Nrf2 to induce sorafenib related ferroptosis

**Figure 4:**
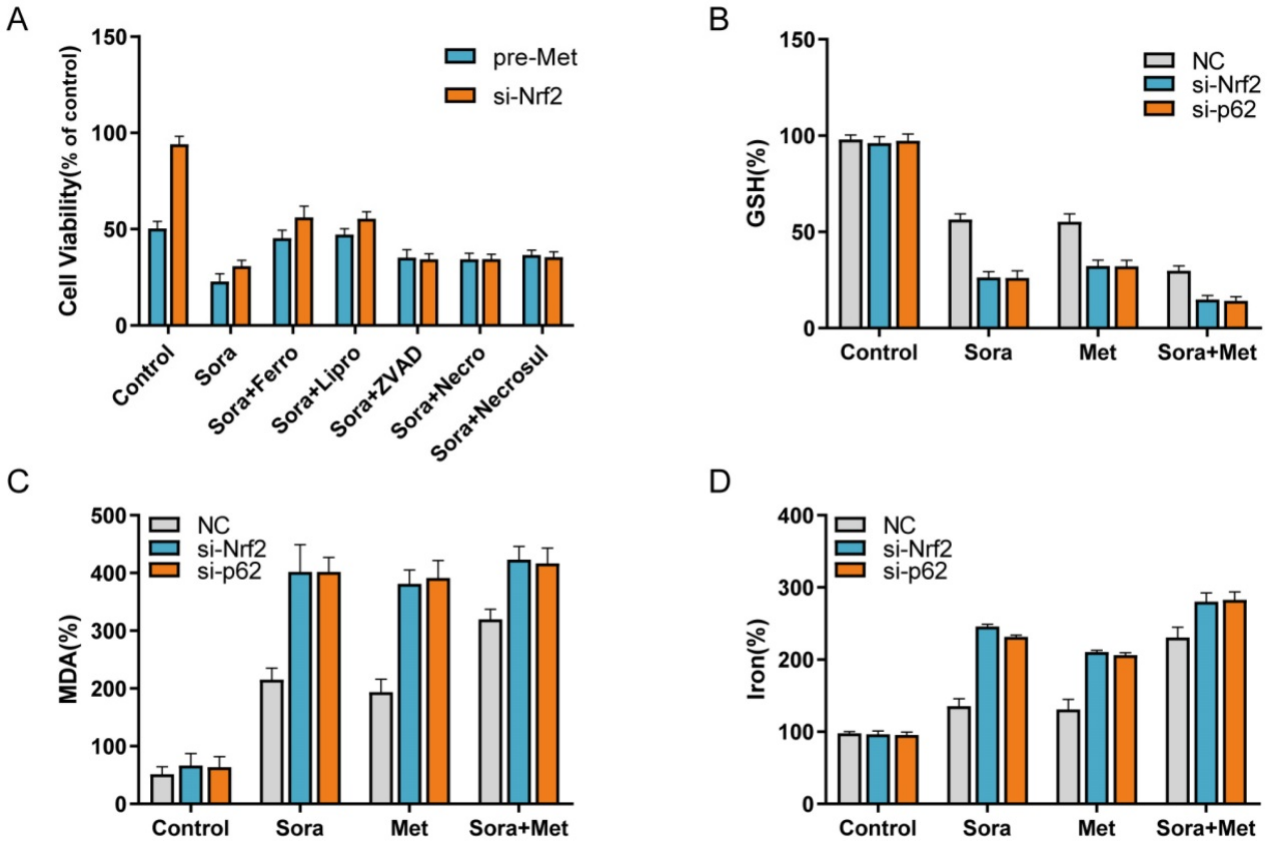
** A** Nrf2 knockdown HCC cells were treated with metformin (15 mM) and sorafenib (10 uM) with or without indicated inhibitors (ferrostatry-1, 1 uM; liproxstatin-1, 100nM; ZVAD-FMK, 10uM; necrostatin, 10uM; necrosulfonamide, 0.5 uM) for 24 hours, and cell viability was assayed (n=3, *p<0.05). **B** Knockdown HCC cells were treated with metformin (15 mM) and sorafenib (10 uM) for 24 hours and GSH level was assayed (n=3, *p<0.05). **C** Knockdown HCC cells were treated with metformin (15 mM) and sorafenib (10 uM) for 24 hours and MDA level was assayed (n=3, *p<0.05). **D** Knockdown HCC cells were treated with metformin (15 mM) and sorafenib (10 uM) for 24 hours and iron level was assayed (n=3, *p<0.05).

Nrf2 is the central role for oxidative stress related cell death, such as apoptosis, necrosis and ferroptosis. To determine whether the combination treatment suppressed HCC cells proliferation through inducing ferroptosis, Nrf2 knockdown group and pre-treated with metformin group were included in the study. Sorafenib-mediated cell death in these two groups was blocked by ferrostatin-1 and liproxstatin-1 (potent ferroptosis inhibitors), while not by ZVAD-FMK, necrostatin and necrosulfonamide (Figure 4A). Lipid peroxidation is the main result for ferroptosis to induce cell death. Compared with control group, the end products of lipid peroxidation, such as MDA, were significantly increased in combination treatment group and Nrf2 knockdown group (Figure 4C), which indicated increased ferroptotic events in HCC cells. At the same time, GSH depletion (Figure 4B) and increase of iron levels (Figure 4D) also indicated the increased ferroptotic events in Nrf2 knockdown group and combination treatment group. These findings suggested the combination treatment suppressed HCC cells proliferation through inhibiting Nrf2 to induce sorafenib related ferroptosis.

### Combination treatment enhanced ferroptosis through Suppression of Nrf2 *in vivo*

**Figure 5:**
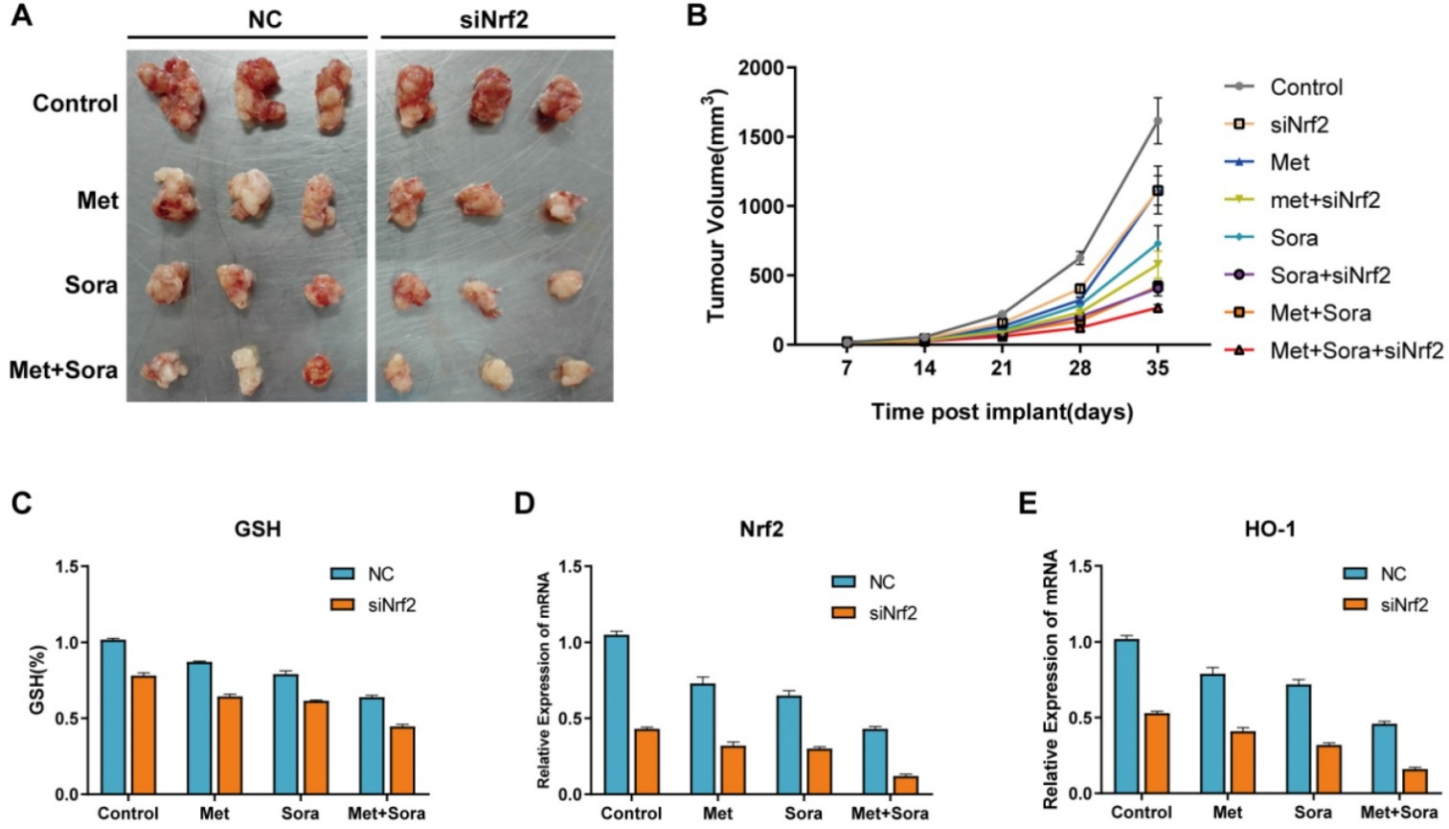
** The combination treatment suppresses proliferation of HCC cells through inhibiting Nrf2 expression *in vivo*. A** Representative image of xenograft-bearing tumors on day 35. **B** Balb/c nude mice were subcutaneously grafted with 1*10^6^ HepG2/Nrf2 knockdown HepG2 cells. Treatment with metformin, sorafenib or the combination was initiated on day 7. Tumor volume was calculated weekly.** C** GSH level analysis of the indicated gene expression in isolated tumor at day 35. **D** qRT-PCR analysis of the Nrf2 expression in isolated tumor at day 35. E qRT-PCR analysis of the HO-1 expression in isolated tumor at day 35. Data represents mean ± standard error (n=5-8 mice/group, p<0.05).

Xenografts were finished by implanting Nrf2 knockdown HCC cells and normal HCC cells into subcutaneous space of the right flank of mice to determine the mechanism whether combination treatment enhanced ferroptosis through suppression of Nrf2 *in vivo*. These mice were treated with sorafenib and metformin at day 7. The results showed co-treated with sorafenib and metformin could effectively reduce the size of tumors compared with control group. Nrf2 knockdown cells had better reaction to sorafenib and metformin compared with normal HCC cells (Figure 5A). Tumor volume was calculated weekly and recorded in Figure 4B. qRT-PCR analysis of the expression of Nrf2 and HO-1 showed combination treatment group significantly inhibited Nrf2 and HO-1 expression (Figure 5D, E) with increased GSH depletion (Figure 5C). These results indicated combination treatment suppressed proliferation of HCC cells *in vivo* through inducing ferroptosis. Inhibiting Nrf2 expression was the possible mechanism for combination treatment in the study.

## Discussion

Hepatocellular carcinoma remains one of the most lethal diseases with poor 5-year survival rate. A large proportion of patients with HCC are diagnosed with advanced stage disease because of nonspecific symptoms in its early stage. Traditional methods for treating HCC only provide a limited improvement in the prognosis. Sorafenib resistance (both primary and acquired) is the main reason for the poor prognosis of HCC patients. Right now, some new therapeutic strategies focus on developing novel targeted therapy to overcome drug resistance 26. Ferroptosis is a new type of iron dependent non-apoptotic cell death found and named by Dixon et al in 2012. A growing number of studies indicate ferroptosis is a promised approach to overcome traditional drug resistance for cancer treatment because of the distinct mechanism compared with other types of cell deaths 27. Metformin is demonstrated to sensitize different cancer cell types to anticancer treatments through inhibiting Nrf2 expression. The intrinsic mechanism is still controversial. In this study, we demonstrated combination treatment of sorafenib and metformin could significantly suppress the proliferation of HCC cells through inhibition of the p62-Keap1-Nrf2/HO-1 pathway *in vitro* and *in vivo*. Ferroptosis is the main cell death type in the combination treatment.

Ferroptosis is a new type of cell death modes through increasing iron-dependent accumulation of lipid ROS, which can be pharmacologically inhibited by lipid peroxidation inhibitors, such as ferrostatin and liproxstatin. Compared with other types of cell death influenced by caspases activity directly or indirectly, ferroptosis seems to have little known molecular cross-talk to other types of cancer death 9. The emerging evidences up to now indicate patients could still get benefits from ferroptosis induction treatment who failed in apoptosis and necroptosis induction treatment 10. Nrf2 is a critical antioxidant transcription factor and plays central role in the pathway of P62-Keap1-Nrf2. Nrf2 could bind to the AREs in their promoters and activate multiple antioxidant enzymes to against oxidative stress. The Nrf2 signaling pathway also plays a central role in sorafenib resistance and ferroptosis induction through HO-1 induction. In this study, we focused on the effects of sorafenib and metformin on the Nrf2 signaling pathway and ferroptosis induction. Metformin suppressed Nrf2 translocation, therefore resulting in decreased HO-1 expression and sensitizing sorafenib for HCC cells. The result showed the combination treatment for HCC cells could induce ferroptosis through P62-Keap1-Nrf2 antioxidative signaling pathway. P62, as the upstream of the pathway, could prevent Nrf2 degradation and increase subsequent Nrf2 nuclear accumulation through inhibition of Keap1 expression. P62 knockdown HCC cells had similar expression of Keap1, Nrf2 and HO-1 and similar reaction to combination treatment compared with Nrf2 knockdown HCC cells, which indicated p62-Keap1-Nrf2 was the main signaling pathway in the combination treatment. Nrf2 could increase the expression of HO-1, which could mediate antiferroptosis activity. Suppression of HO-1 expression though combination treatment and Nrf2 knockdown significantly increased ferroptosis in HCC cells, indicating that ROS accumulation is extremely important in the development of ferroptosis.

## Conclusions

**Figure 6:**
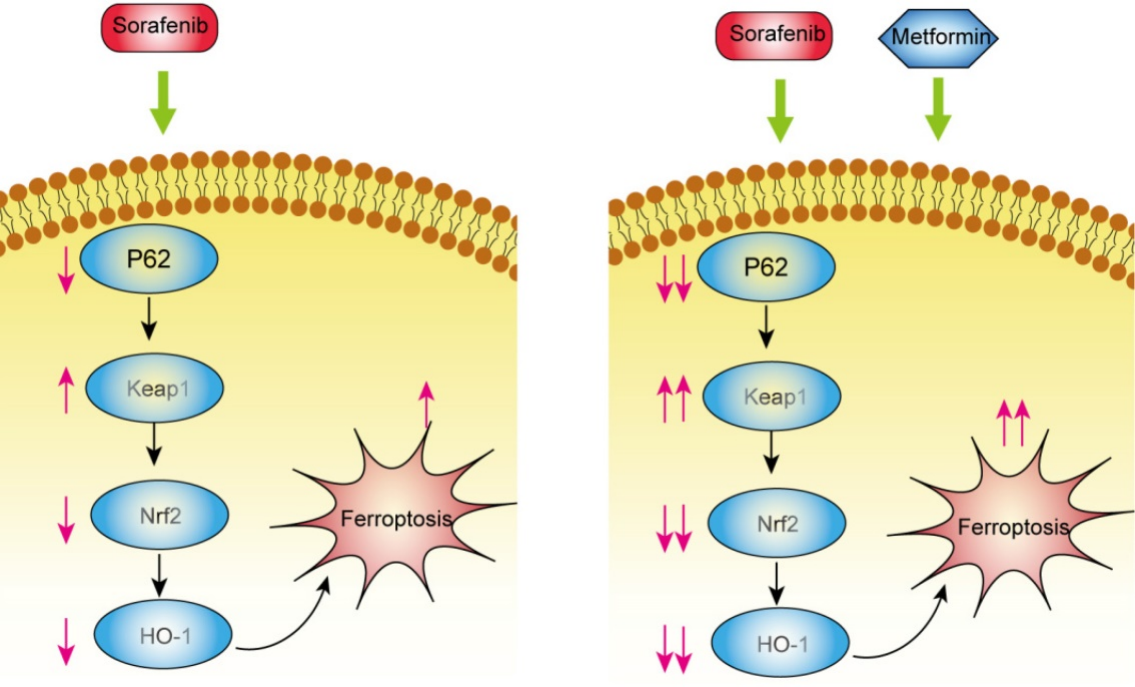
Combination of metformin and sorafenib induces ferroptosis of hepatocellular carcinoma through p62-Keap1-Nrf2 pathway.

Metformin inhibited Nrf2 expression and augmented the cytotoxic effects of sorafenib through inhibiting p62-Keap1-Nrf2 signaling pathway *in vitro* and *in vivo* (Figure 6). Combination treatment increased intracellular ROS production and ferroptosis induction and inhibited tumor growth *in vivo*.

## Figures and Tables

**Table 1:** Clinico-pathological correlation with Nrf2 expression in HCC patients

Clinical Features	Total No. of cases	Nrf2 (High)	Nrf2 (Low)	*P* value
**Gender**				
Male	32	17 (53.1%)	15 (46.9%)	0.556
Female	18	8 (44.4%)	10 (55.6%)	
**Age**				
≤59	15	7 (46.7%)	8 (53.3%)	0.758
≥60	35	18 (51.4%)	17 (48.6%)	
**No. of nodules**				
Single	24	11 (45.8%)	13 (54.2%)	0.571
Multiple	26	14 (53.8%)	12 (46.2%)	
**Tumor size**				
<5 cm	33	17 (51.5%)	16 (48.5%)	0.765
≥5 cm	17	8 (47.1%)	9 (52.9%)	
**HBsAg**				
Yes	45	23 (51.1%)	22 (48.9%)	0.637
No	5	2 (40%)	3 (60%)	
**AFP Level**				
<20 ng/ml	12	4 (33.3%)	8 (66.7%)	0.185
≥20 ng/ml	38	21 (55.3%)	17 (44.7%)	
**TNM**				
Stage I/II	23	10 (43.5%)	13 (56.5%)	0.395
Stage III/IV	27	15 (55.6%)	12 (44.4%)	
**Differentiation**				
Well/Moderate	28	13 (46.4%)	15 (53.6%)	0.569
Poor	22	12 (54.5%)	10 (45.5%)	
**Vascular invasion**				
No	34	16 (47.1%)	18 (52.9%)	0.544
Yes	16	9 (56.3%)	7 (43.7%)	
**Microvascular invasion**				
Yes	32	17 (53.1%)	15 (46.9%)	0.556
No	18	8 (44.4%)	10 (55.6%)	

## Abbreviations

HCC: hepatocellular carcinoma
Nrf2: NF-E2-related factor 2
PUFAs: peroxidation of polyunsaturated fatty acids
KEAP1: kelch-like ECH-associated protein 1
ROS: reactive oxygen species
CCK8 kit: Assay kits for the detection of cell survival
HE: hematoxylin eosin
IOD: Integrated Option Density
AOI: Area of Interesting
FBS: fetal bovine serum
MDA: malondialdehyde
